
JOURNAL INFORMATION
==============================
NLM Title Abbreviation: ScientificWorldJournal
Journal ID: ScientificWorldJournal
NLM Journal ID: 101131163
Journal ID: TSWJ

The Scientific World Journal

ISSN: 2356-6140
EISSN: 1537-744X
Publisher: Wiley

ARTICLE INFORMATION
==============================
PMCID: PMC5848736
PMID: 18264634
DOI: 10.1100/tsw.2008.28
Article ID: 487201
Article version: 1
Subjects: Clinical Image

Nephrogenic Fibrosing Dermopathy

Dhungel Ashish *
Lattupalli Rakesh
Topf Joel
Division of Nephrology, St. John Hospital and Medical Center, Detroit, MI, USA
*Ashish Dhungel: ashish.dhungel@stjohn.orgAcademic Editor: Daniel Batlle

Publication date: 2008
Electronic publication date: 2008 Feb 6
Volume: 8
First page: 164
Last page: 165
Received 2007 Dec 12; Accepted 2007 Dec 12
Copyright: Copyright © 2008 Ashish Dhungel et al.
Copyright year: 2008
License: This is an open access article distributed under the Creative Commons Attribution License, which permits unrestricted use, distribution, and reproduction in any medium, provided the original work is properly cited.
License URL: https://creativecommons.org/licenses/by/3.0/

Keywords: nephrogenic fibrosing dermopathy, dialysis, gadolinium

==============================
Clinical Image
*TheScientificWorldJOURNAL* (2008) 8, 164–165
ISSN 1537-744X; DOI 10.1100/tsw.2008.28

TheScientificWorldJOURNAL
www.thescientificworld.com

### Nephrogenic Fibrosing Dermopathy

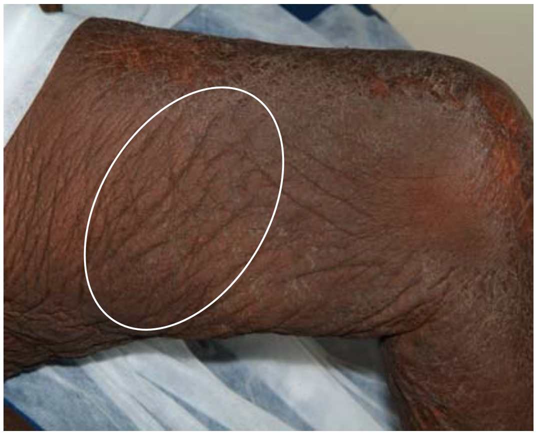

Thickening and puckering of skin commonly affecting the thigh area.

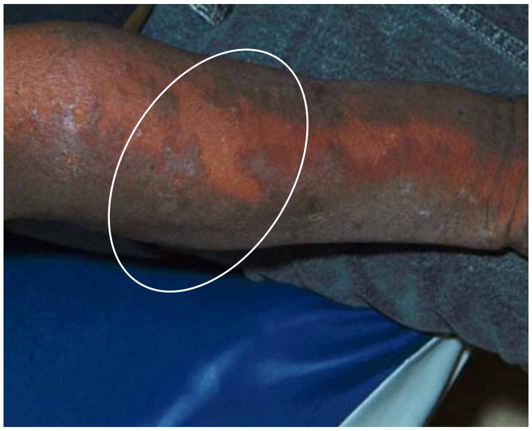

Erythema with skin tightening and hyperpigmented plaques.

Ashish Dhungel*, Rakesh Lattupalli, and Joel Topf
*Division of Nephrology, St. John Hospital and Medical Center, Detroit, MI*

**E-mail:** Ashish.Dhungel@stjohn.org

*Received December 12, 2007; Accepted December 12, 2007; Published February 6, 2008*

**KEYWORDS**: nephrogenic fibrosing dermopathy, dialysis, gadolinium

A 50-year-old, African American female was admitted with acute kidney injury from acute allograft rejection and underwent MRI (magnetic resonance imaging) with gadolinium. Hemodialysis was initiated secondary to graft failure 2 days later. Over the next 30 days, skin tightening was noticed initially in the lower extremities, which gradually progressed to involve the back and upper limbs. Ankle and knee joints developed contractures, leading to immobility. Skin biopsy revealed dermal sclerosis with fibrocytic proliferation, consistent with nephrogenic fibrosing dermopathy (NFD). NFD is an uncommon, but increasingly recognized, skin condition observed in patients with renal failure who were exposed to gadolinium.

### REFERENCES

1. Cowper, S.E., Su, L., Robin, H., Bhawan, J., and LeBoit, P.E. (2001) Nephrogenic fibrosing dermopathy. *Am. J. Dermatopathol.* **23,** 383–393.

*Corresponding author.
©2008 with author.
Published by TheScientificWorld; www.thescientificworld.com

2. http://www.fda.gov/cder/drug/advisory/gadolinium_agents_20061222.htm [U.S. Food and Drug Administration. Center for Drug Evaluation and Research. Public Health Advisory. Update on Magnetic Resonance Imaging (MRI) Contrast Agents Containing Gadolinium and Nephrogenic Fibrosing Dermopathy.

**This article should be cited as follows:**

Dhungel, A., Lattupalli, R., and Topf, J. (2008) Nephrogenic fibrosing dermopathy. *TheScientificWorldJOURNAL* **8**, 164–165. DOI 10.1100/tsw.2008.28.
